# Longer duration entry mitigates nystagmus and vertigo in 7-Tesla MRI

**DOI:** 10.3389/fneur.2023.1255105

**Published:** 2023-11-16

**Authors:** Jacob M. Pogson, Ari Shemesh, Dale C. Roberts, David S. Zee, Jorge Otero-Milan, Bryan K. Ward

**Affiliations:** ^1^Department of Neurology, The Johns Hopkins University School of Medicine, Baltimore, MD, United States; ^2^Department of Neurology, Royal Prince Alfred Hospital, Camperdown, NSW, Australia; ^3^Department of Ophthalmology, Hadassah Medical Center, Jerusalem, Israel; ^4^Herbert Wertheim School of Optometry and Vision Science, University of California, Berkeley, Berkeley, CA, United States; ^5^Department of Neuroscience, The Johns Hopkins University, Baltimore, MD, United States; ^6^Department of Otolaryngology-Head and Neck Surgery, The Johns Hopkins University School of Medicine, Baltimore, MD, United States; ^7^Wilmer Eye Institute, The Johns Hopkins University, Baltimore, MD, United States

**Keywords:** vertigo, nystagmus, mathematical model, MRI, safety, Lorentz force, magnetohydrodynamics

## Abstract

**Introduction:**

Patients and technologists commonly describe vertigo, dizziness, and imbalance near high-field magnets, e.g., 7-Tesla (T) magnetic resonance imaging (MRI) scanners. We sought a simple way to alleviate vertigo and dizziness in high-field MRI scanners by applying the understanding of the mechanisms behind magnetic vestibular stimulation and the innate characteristics of vestibular adaptation.

**Methods:**

We first created a three-dimensional (3D) control systems model of the direct and indirect vestibulo-ocular reflex (VOR) pathways, including adaptation mechanisms. The goal was to develop a paradigm for human participants undergoing a 7T MRI scan to optimize the speed and acceleration of entry into and exit from the MRI bore to minimize unwanted vertigo. We then applied this paradigm from the model by recording 3D binocular eye movements (horizontal, vertical, and torsion) and the subjective experience of eight normal individuals within a 7T MRI. The independent variables were the duration of entry into and exit from the MRI bore, the time inside the MRI bore, and the magnetic field strength; the dependent variables were nystagmus slow-phase eye velocity (SPV) and the sensation of vertigo.

**Results:**

In the model, when the participant was exposed to a linearly increasing magnetic field strength, the per-peak (after entry into the MRI bore) and post-peak (after exiting the MRI bore) responses of nystagmus SPV were reduced with increasing duration of entry and exit, respectively. There was a greater effect on the per-peak response. The entry/exit duration and peak response were inversely related, and the nystagmus was decreased the most with the 5-min duration paradigm (the longest duration modeled). The experimental nystagmus pattern of the eight normal participants matched the model, with increasing entry duration having the strongest effect on the per-peak response of nystagmus SPV. Similarly, all participants described less vertigo with the longer duration entries.

**Conclusion:**

Increasing the duration of entry into and exit out of a 7T MRI scanner reduced or eliminated vertigo symptoms and reduced nystagmus peak SPV. Model simulations suggest that central processes of vestibular adaptation account for these effects. Therefore, 2-min entry and 20-s exit durations are a practical solution to mitigate vertigo and other discomforting symptoms associated with undergoing 7T MRI scans. In principle, these findings also apply to different magnet strengths.

## Introduction

Vertigo, dizziness, and imbalance are commonly reported by patients and technologists when near high-field strength magnets (>4 Tesla, T) used for magnetic resonance imaging (MRI) (1–5), including one extraordinary complication of vomiting immediately after exiting a 3T MRI machine with a subsequent post-surgical CSF leak (6). A study of 573 participants undergoing a 7T MRI found that 60% reported “vertigo” during bed motion (duration not described) and 32% when in the isocenter of the MRI scanner (7). Another study of 102 participants who were slowly moved in and out (duration not described) of a 7T MRI found that 25% of subjects reported their vertigo as tolerable and 5% as unpleasant (1), with four participants terminating participation early due to intolerable nausea. Nevertheless, as these symptoms are usually transitory, the majority of humans tolerate 7T MRI scans (1, 8).

Magnetic vestibular stimulation (MVS) of the inner ear explains these symptoms. In the normal state, the inner ear has a constant electric current flowing from the dark cells to the hair cells of the utricular macula through the potassium-enriched endolymph. This electric current drives the exquisitely sensitive response of the utricle to *linear* accelerations (9). Near the utricular macula are the lateral and superior semicircular canal cupulae (SCC), which are exquisitely sensitive to *angular* accelerations (10). Inside an MRI scanner, the electric current entering the utricular macula in each ear interacts with the MRI static magnetic field to create a Lorentz (magneto-hydrodynamic [MHD]) force in the endolymph that pushes on the cupulae of the nearby semicircular canals (11–13). The force scales linearly with magnetic field strength (11, 13). Thus, when a human with an intact vestibular system lies in a 7T MRI magnet, the Lorentz force causes the endolymph to push on the cupulae, changing the activity of the angular vestibulo-ocular reflex (VOR) pathway, generating both a transient sensation of motion and a sustained beating of the eyes (nystagmus), with alternating slow phases from the VOR and quick phases that reset the position of the eye (11, 14, 15).

Previous studies found that in strong MRI scanners, slow-phase eye velocity (SPV) of nystagmus scales with the strength of the magnetic field (11) and with the pitch angle of the head in the magnetic field (11, 16). The nystagmus partially adapts over time (11, 17), presumably through a combination of peripheral and central adaptation mechanisms. Upon exiting the MRI, there is an aftereffect that reflects the adaptation that has taken place in the MRI, in which the direction of nystagmus and the sensation of rotation transiently reverses (17, 18). A mathematical model and additional human experiments found that three operators in series with increasingly long-time constants explain the decay in nystagmus and the aftereffect (17).

Several studies on vertigo during MVS report that perceptual thresholds are higher than nystagmus SPV thresholds. Mian et al. (15) reported on 25 participants that entry durations of 40–60 s into a 7T MRI scanner evoked a rotational percept in 86% and a translational percept in 25%, including tilting. The onset of these perceptions was always at a greater magnetic field strength than the onset of nystagmus (y-intercept of 3.3 T) and the magnetic field strength at which perceptions and nystagmus began were strongly correlated (R^2^ = 0.72, *p* < 0.01). With a similar experimental protocol, Mian et al. (16) then studied the effect of head position in the pitch and roll planes, reporting significant effects of head pitch angle on the perceived speed and duration of rotation and the magnetic field at which the rotation began, and then head roll on nystagmus SPV, concluding that they share a common peripheral vestibular origin. These MVS studies are consistent with traditional rotational stimuli studies, which report that the perceptual threshold is higher for angular accelerations in the yaw plane than in the recordable nystagmus SPV (19–21). However, all of these studies used much shorter angular rotations (<2 min) than typical MVS studies (>2 min).

Given the evidence to support a common source of nystagmus and vertigo to MVS, we hypothesized that a technique that blunted the peak in SPV of nystagmus might also diminish the sensation of vertigo. Here, we developed and tested an MVS and vestibular adaptation model to find a simple solution for vertigo and dizziness in high-field MRI machines.

## Materials and methods

To optimize the acceleration and the speed of entry into and exit from the MRI static magnetic field, we first created a linear control systems model of the direct and indirect VOR pathways, including linear and angular pathways and adaptation mechanisms (17, 22–24). We then experimentally recorded nystagmus and the subjective experience of eight individuals in a 7T MRI scanner (Philips Achieva 7 Tesla, Philips Research, Hamburg, Germany) while undergoing the conditions suggested by the model simulations. Experiments were approved by the Johns Hopkins Institutional Review Board (IRB NA_00041628), and informed consent was obtained from all participants.

### Mathematical model

We modeled the VOR with a 3D control system based on an earlier study (17, 22, 23), which included elements for vestibular adaptation operators (Tc = 80 s, 300 s, and 3000 s), a central velocity storage integrator (Tc = 16 s), and two cross-products for tilt estimation and rotational feedback in a similar structure as an earlier study (24).

The simulation included 300 s of MVS exposure. The Lorentz force (***F***) is represented by ***F*** = ***Lj*** x ***B***, where ***j*** represents current density, ***B*** is the static magnetic field, **L** is the distance over which the current flows, and x is a cross-product that follows the right-hand rule (11). Since the Lorentz force scales with magnetic field strength, we represented different magnetic field strengths as multiples of the baseline acceleration stimulus. All data were modeled for the head in the supine position. The response was SPV of nystagmus. We modeled the stimulus as a constant acceleration ramp (17).

### Variables

**Figure 1:**
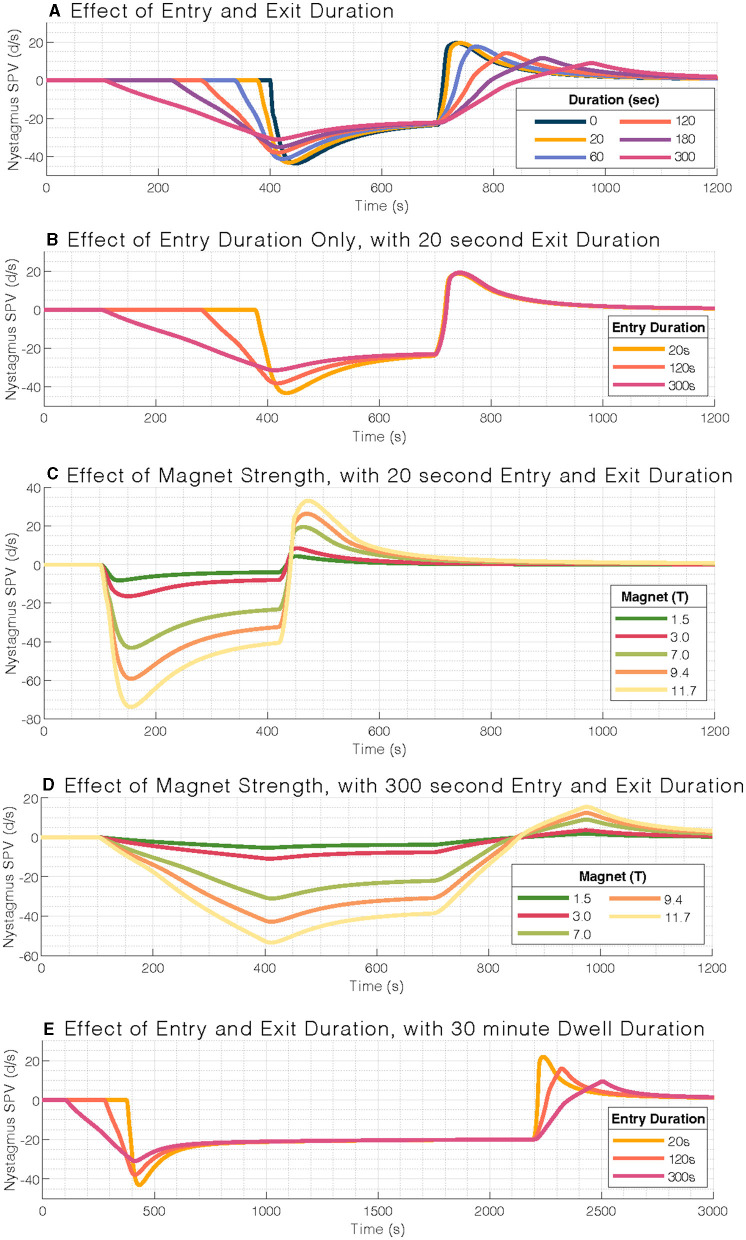
Mathematical model output of nystagmus slow-phase eye velocity (SPV). **(A)** Effect of entry and exit duration on nystagmus slow-phase velocity (SPV). **(B)** Entry durations only affect the per-peak response and not the post-peak response (set at 20 s). **(C)** Effect of magnetic field strength on nystagmus slow-phase velocity for a typical 20-s entry and exit duration, with a scaling response. **(D)** Effect of magnetic field strengths for a 300-s entry duration. **(E)** Similar pattern of blunted nystagmus response to **(A)**, but at 30-min stimulus duration. Note the longer timescale for **(E)**. Simulations within each panel are aligned to the stimulation period.

The independent variables in the model included the duration in the MRI bore (5 and 30 min), magnet strength (1.5, 3.0, 7.0, 9.4, and 11.7 T), and the duration of time required to enter and exit the MRI bore (0, 20, 60, 120, 180, and 300 s). Variable combinations deemed of clinical interest were selected for visualization (Figure 1). The durations and the MRI magnet strengths were chosen to reflect current and future research and medical practice scenarios.

The dependent variable was the nystagmus SPV in degrees per second (°/s) and the participant's report of subjective sensations. The 0-s entry/exit duration was used to model a participant who enters the MRI bore with their head positioned in their individual MVS null position, which generates no vertigo or nystagmus (11). Once the participant is in the 7T magnetic field, they can pitch their head to their natural rest position, resulting in a near 0-s latency onset of MVS. The 20 s duration reflects the time it takes for the subject to enter the 7T MRI bore completely using the standard motorized entry provided by the MRI manufacturer. Additional entry and exit durations were chosen based on an earlier study that found that adaptation of the nystagmus in the MRI decays exponentially (17).

### Experiment

In eight healthy volunteers (seven men, 24 to 63 years; four naive to MVS), we recorded 3D binocular eye movements in darkness using infrared video goggles (RealEyes, MicroMedical Technologies, Inc.) that had attached a 3D accelerometer and a 3D magnetometer (12). The accelerometer determined the participant's head pitch to gravity, and the magnetometer recorded the magnetic field strength next to the participant's right temple. Custom software extracted the position of both eyes, the position of the head, and the strength of the magnetic field. The amplitude of the peak SPV was recorded after entry (“per-peak” period) and following exit (“post-peak” period) from the MRI. The mean SPV was also calculated over 30 s before entry into the MRI (“baseline” period) and 30 s before exit from the MRI bore (“per-steady” period).

Participants underwent three conditions during a single testing session, entering and exiting the 7T MRI bore over 20 s, 120 s, and 300 s durations. For example, for the 20-s trial, eye movements were recorded outside the bore for 2 min of baseline, for the entry duration of 20 s into the bore, at the isocenter for 5 min, for the exit duration of 20 s, and outside the bore again for 4 min, and for a total of 11 min and 40 s. Entry and exit durations for the 120 s (2 min) and 300 s (5 min) trials were 15 and 21 min, respectively. Participants were asked to describe the nature and intensity of their vestibular perception and when the sensations ceased. We calculated perception duration and then categorized the sensations as spinning vertigo, tilting, or non-specific “dizziness” with an intensity of none, low, moderate, or high (as an ordinal scale). Experiments used the static MRI magnetic field alone; no images were obtained. The order of the three trials was randomized for each participant.

The MRI was entered using the standard 20-s duration for the motorized table (10.8 cm/s over 2 m travel). The constant velocity motion of the motorized table through the non-linear magnetic field gradient creates non-linear changes in the Lorentz force. To reflect the conditions in the model, we needed to increase the force linearly over time for the longer trials. The bed was controlled manually for entry over 120 s and 300. We aimed to enter the bore linearly to the magnetic field, accounting for the magnetic field gradient. This requires adjusting the rate of speed of the table because of the non-linearity of the gradient in the fringe magnetic field. Using the magnetometer, we first mapped the magnetic field from the end of the table where the head would lie to the middle of the bore from 1 to 7 T, marking a distance for each Tesla. The table was disconnected from the motor and then manually pushed in and pulled out of the bore at a rate of 17 s/T for the 120-s entry/exit duration (~0.05 T/s) and 42 s/T for the 300-s entry/exit duration (~0.02 T/s). See Table 1 for more values. The magnetic field strength was also monitored in real time throughout the experiment.

**Table 1:** Numerical values for each duration (20, 120, 300 s) in two paradigms: motion at a constant Tesla rate (T/sec) or a constant table velocity (cm/sec).

**Paradigm**	**Mean rate**	**Duration (sec)**		
		**20**	**120**	**300**
Constant tesla	Tesla/sec	0.616	0.052	0.022
	cm/sec	12.500	1.040	0.446
Constant bed velocity	Tesla/sec	0.510	0.049	0.020
	cm/sec	10.800	1.040	0.417

The longer duration conditions show similar rates between paradigms and should be similarly effective.

We used the same video goggles system as a control experiment to record eye movements from six age-matched healthy controls for 11 min while lying supine in a separate laboratory environment away from the MRI machine (only the earth magnetic field).

### Statistical analysis

We used the R software (v3.5.1 “Feather Spray”) and packages (including tidyverse, broom, emmeans, and ordinal) to analyze and plot data (25–28). Simple linear models were used to analyze continuous experimental data, including nystagmus SPV and vertigo (lm:stat v3.5.1). We used a subject-dependent variable to account for non-independent responses because there were only eight participants, e.g., *SPV* ~ *ConditionDuration* + *Subject*. Cumulative link mixed models were used for ordinal experimental data, including vertigo intensity rating (clmm::ordinal v2019-12-10). An alpha of 0.05 was used to indicate “statistical significance.”

## Results

### Mathematical model

We first examined the effect of entry and exit durations on nystagmus SPV in a 7T MRI. The model iterated through six entry and exit durations: 0, 20, 60, 120, 180, and 300 s (Figure 1A). The per-peak (after entering the MRI bore) and post-peak (after exiting the MRI bore) amplitude of the eye movement response was reduced with increasing duration of entry and exit, respectively, in a strongly linear relationship (adjusted R^2^ = 0.99 and R^2^ = 0.96, respectively).

The per-peak amplitudes were larger than the post-peak amplitudes. We also studied the effect of different entry durations followed by the standard 20-s exit duration (Figure 1B); the post-peak response was not affected by the time of entry into the MRI bore.

We then examined the effect of magnetic field strength to simulate common clinical MRI strengths (1.5T, 3T, and 7T) and potential future clinical field strengths (9.4T, 11.7T, Figure 1C). The nystagmus response is expected to increase with stronger magnetic field strengths. We found a linear relationship with amplitude, including at 300-s entry duration (Figure 1D). Over the range of stimulus durations modeled, *i.e.*, the time inside the MRI bore, the amplitude of the post-peak response was only minimally affected (Figure 1E).

In summary, we found a relationship between the duration of entry/exit modeled and peak nystagmus response, such that the 300-s (5-min) entry duration best mitigated the nystagmus SPV in a 7T MRI scanner (Figure 1A). A possible trade-off between practicality and minimizing the response amplitude is that one could use a 120-s (2-min) entry duration and a 20-s exit duration (Figure 1B). Note that the entry into the MRI bore generates a greater peak SPV response than the exit, and the post-peak SPV response is independent of the per-peak response.

### Experiment

**Figure 2:**
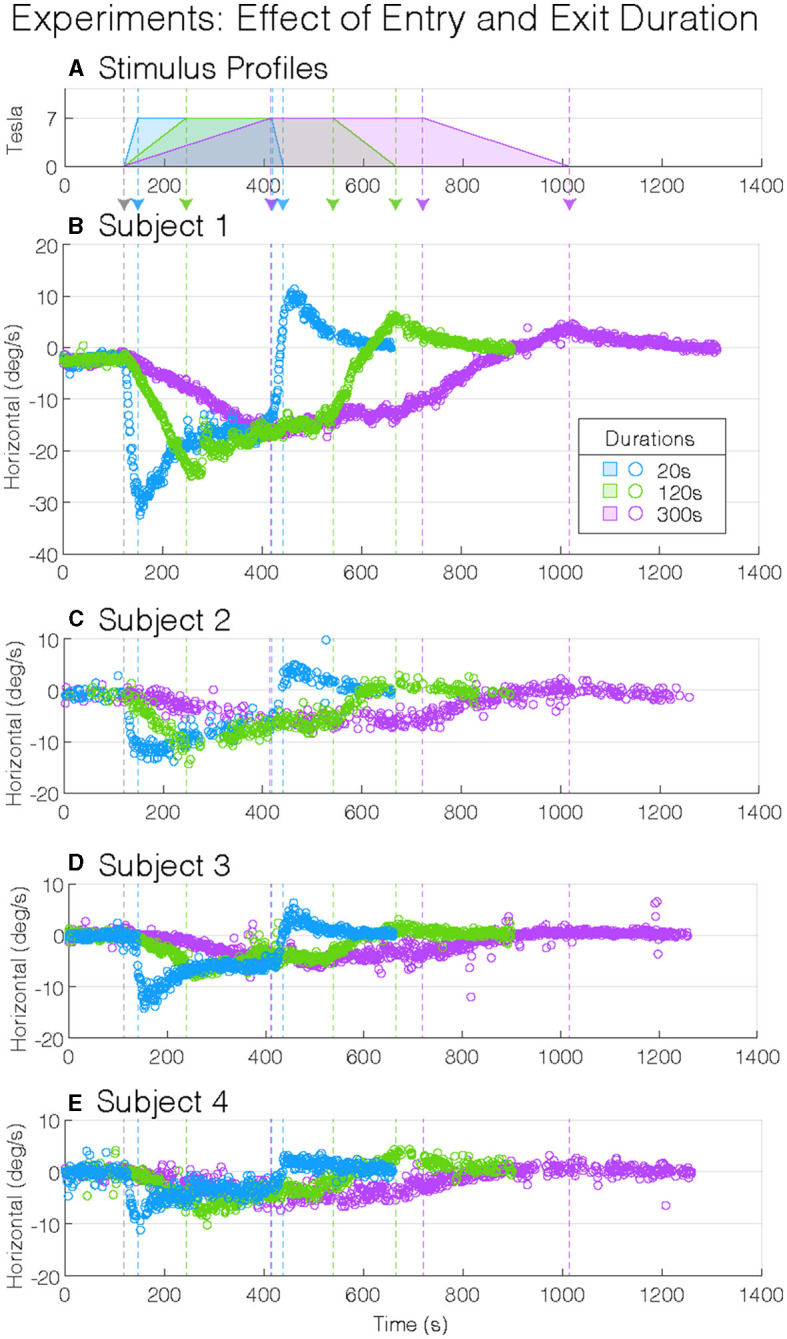
Experimental magnetic vestibular stimulation nystagmus slow-phase eye velocity (SPV). Stimulus profile **(A)** and subject nystagmus SPV responses from four subjects **(B–E)** to three entry and exit durations (20, 120, and 300 s: blue, green, and purple). Each nystagmus SPV response profile follows the corresponding stimulus profile, with the entry and exit peak SPV greater to shorter durations. Although the four participants showed variation in their peak SPV amplitudes, they showed similar general response patterns: longer durations of entry into and exit from the magnet decrease the amplitude of the peak SPV. The vertical-colored dashed lines (—) indicate changes in stimulus amplitudes **(A)**.

We then experimentally tested the mathematical model in eight healthy participants, of which four were naïve to MVS (Figure 2).

**Figure 3:**
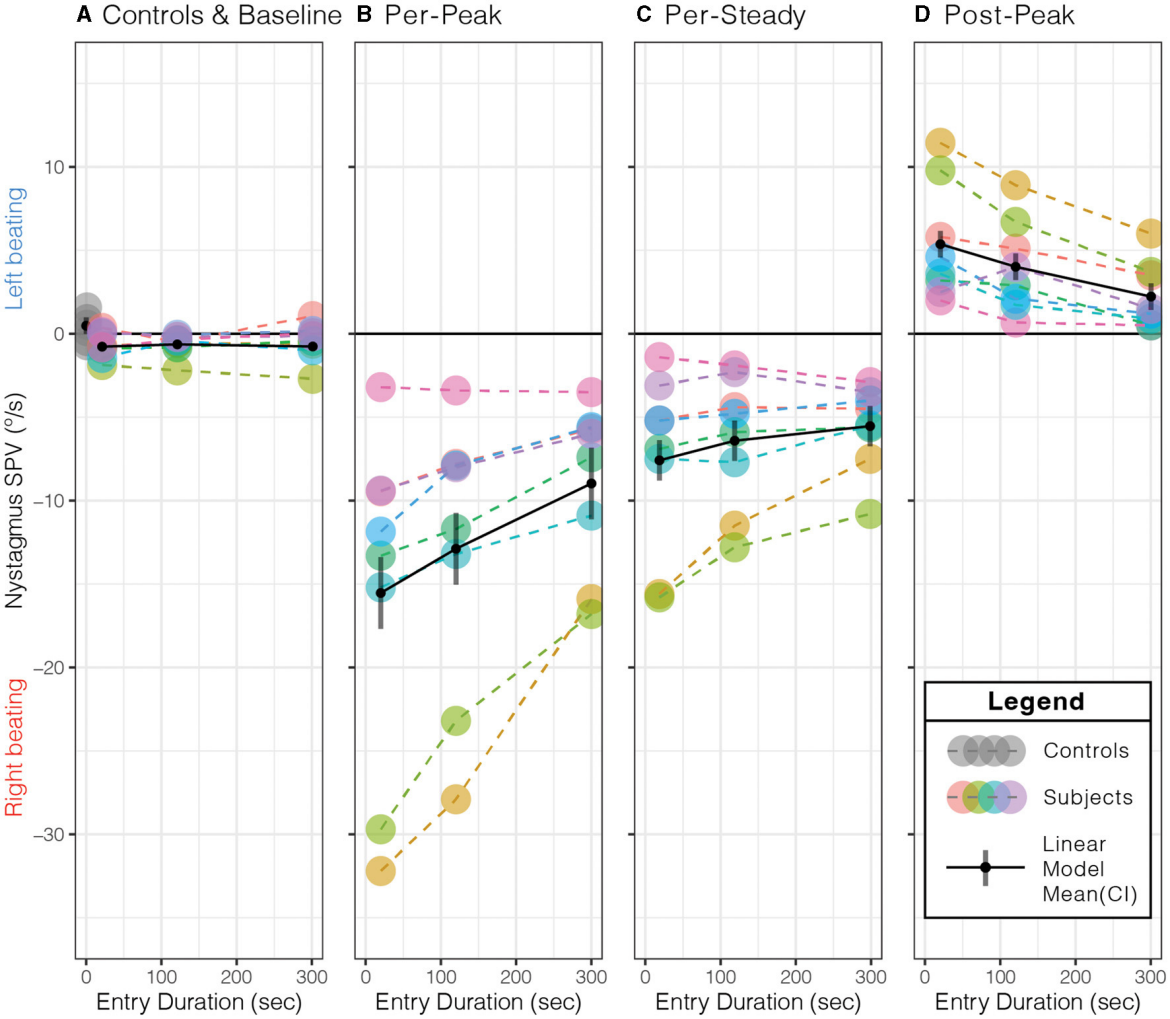
Effect of entry duration on peak slow-phase eye velocity (SPV) responses. **(A)** Compared to control subjects away from the MRI field at the baseline, subjects' nystagmus SPV was right beating at a similar SPV (*p* = 0.13). Longer entry durations showed a decrease in the **(B)** per-peak SPV just after entering the MRI bore, **(C)** per-steady SPV just before exiting the MRI bore, and **(D)** post-peak SPV just after leaving the MRI bore. The per- and post-peaks showed a duration effect (*p* ≤ 0.0014, Table 2). Each color represents the same subject across all panels, with dashed lines (- - -) connecting each data point (•) within each panel. The solid black lines (—–) represent the simple linear model's mean (•) and 95 % confidence interval (CI). See Table 2 for the simple linear model's mean (95% CI) values for each panel.

During the 2 min baseline period before entering the MRI, while in the fringe magnetic field (~1T), the horizontal nystagmus SPV was similar for all participant test sessions (mean(CI), −0.6(−0.89–−0.22) °/s, *p* = 0.56, Table 1), i.e., slight right-beating nystagmus (Figure 3A). The mean MVS horizontal nystagmus SPV was in the opposite direction—right beating rather than left beating—compared to six normal controls away from the magnet (difference (SEM), −1.0(0.35) °/s, *p* = 0.0087), although the absolute amplitude was similar [mean(SEM), 0.6(0.35) °/s, *p* = 0.13, Figure 3A]. Thus, the MRI fringe magnetic field biased the direction but not the amplitude of any spontaneous horizontal nystagmus the participants may have had when lying outside the MRI bore. The order sequence of entry conditions on subsequent baseline trial periods did not show an effect (*p* = 0.53).

As expected in our MRI scanner, all subjects showed a right-beating nystagmus while in the magnetic field (Figure 3B). Similarly, as predicted from the model, the per-peak SPV response was affected by the duration of entry; the 20-s condition showed the greatest peak SPV at the mean (lower–upper 95% CI) to −15.5(−17.7–−13.4)°/s, followed by the 120-s condition at −12.9(−15.0–−10.7)°/s and the 300-s condition at −9.0(−11.1–−6.8)°/s (Figure 3B, Table 2, *p* = 0.0014). The model showed a strong correlation (adjusted R^2^ = 0.88).

**Table 2:** Estimated marginal mean (95% confidence interval, CI) values from the simple linear model of each condition and period that correspond to the panels of Figure 3.

**Period**	**Condition**	**Model mean (95% CI)**	**P-value**
Controls	—	0.48 (−0.02–0.98)	—
Baseline	20	−0.7 (−0.98–−0.34)	0.5602
	120	−0.6 (−0.96–−0.32)	
	300	−0.5 (−0.77–−0.13)	
Per-peak	20	−15.5 (−17.69–−13.39)	*0.0014*
	120	−12.9 (−15.04–−10.74)	
	300	−9.0 (−11.12–−6.83)	
Per-steady	20	−7.6 (−8.79–−6.38)	0.0651
	120	−6.4 (−7.62–−5.21)	
	300	−5.5 (−6.74–−4.33)	
Post-peak	20	5.4 (4.57–6.16)	*0.0001*
	120	4.0 (3.22–4.81)	
	300	2.2 (1.43–3.02)	

The p-value is the main effect of each period.

*Post-hoc* pairwise comparisons showed that the entry duration affected the per-peak amplitude of the SPV (Figure 3B). Compared to the 20-s duration, the 300-s condition was 6.6 (1.42)°/s or 42.2% less (*p* = 0.0011), while the 120-s condition was 2.6 (1.42)°/s or 17.1 % less but was not different (*p* = 0.18). The 300-s condition was 3.9 (1.42)°/s or 30.4% less than the 120-s condition (*p* = 0.038).

In contrast, the nystagmus SPV just before exiting the MRI after 5 min of exposure, averaged over the 30 s before exit, only showed weak evidence that duration of entry affected the SPV (*p* = 0.0651), with a mean (CI) of −6.5(−8.72–−4.31)°/s of right-beating nystagmus for all subjects and conditions (Figure 3C, Table 2).

Upon exiting, all participants showed a change of direction to left-beating nystagmus (Figure 3D, Table 2). The exit duration showed a strong main effect on the post-peak SPV amplitude (*p* = 0.0001). *Post-hoc* pairwise comparisons showed that compared to the 20-s condition, the 300-s condition was 3.1(0.52)°/s or 58.6 % less (*p* = 0.0001), while the 120-s condition was 1.4(0.52)°/s or 25.3% less but was not different (*p* = 0.053) (Figure 3D). The 300-s condition was 1.8(0.52)°/s or 44.5 % less than the 120-s condition (*p* = 0.011).

In summary, the MRI entry and exit duration affected the peak nystagmus SPV, but the largest effect was on entry. After both entry and exit, the 300-s condition showed the largest effect, as the 20-s and 120-s conditions were similar. After 5 min of exposure to the magnet, the effect of entry duration was no longer measurable.

Participants were encouraged throughout the experiment to report any vestibular sensations. During entries and exits, participants were asked to describe the direction, intensity, and duration of any spinning vertigo, non-specific dizziness, or static tilting. At baseline, all eight subjects reported no spinning, tilting, or dizziness sensations (100%). For the 20-s condition, all eight participants reported a mean (SD) of 79 (63.4) s of moderate to high-intensity spinning vertigo during entry. During exit, all eight participants reported 46 (27.2) s of spinning vertigo at a low-to-moderate intensity. For the 120-s condition, three participants reported low-intensity tilting sensations (38%) lasting 61 (57.5) s during entry. Then, on exiting, no participant reported spinning vertigo (0%), but two reported tilting (25%) that lasted for 24 and 68 s. For the 300-s condition, one participant reported a “very slow rotation” of low intensity on entry and then again on exit, one reported a low-intensity tilting sensation, and the other six participants reported no vertigo, dizziness, or tilting on entry or exit (75%).

**Figure 4:**
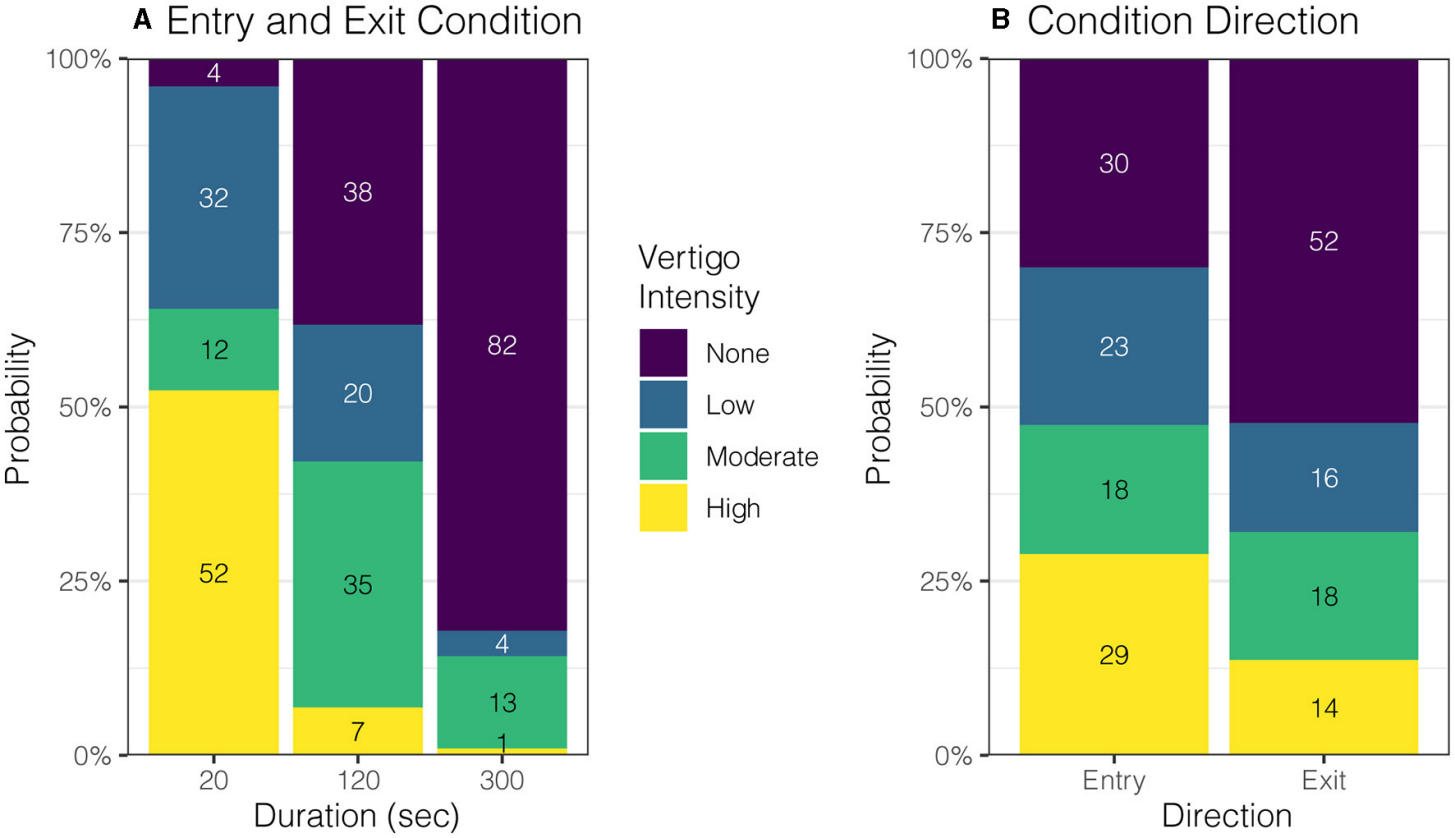
Effect of entry duration and direction on perceived vertigo intensity. **(A)** Shorter duration entry conditions caused higher rated intensity of perceived vertigo or tilting compared to longer duration conditions (*p* = 0.0003). **(B)** Perceived motion intensity was rated higher on entry than exit (*p* = 0.0035).

The entry and exit condition showed a strong effect on perception intensity (Figure 4A, *p* = 0.0003); *post-hoc* comparisons to the 20-s condition found that the 120-s and 300-s conditions showed significantly lower intensity ratings (difference in mean probability (standard error): 1.5 (0.22), 2.4 (0.16), *p* ≤ 0.0001), and the 300-s condition was lower than 120-s condition (0.9 (0.26), *p* = 0.0015). Generally, entering the magnet caused higher intensity ratings compared to exiting the magnet (0.7(0.14), *p* ≤ 0.0001, Figure 4B), which held across all condition durations (*p* = 0.3661). Nystagmus SPV showed a marginal relationship with perceived vertigo intensity (*p* = 0.0495). The mean duration of any motion sensation was 24 (9.4) s shorter when exiting the magnet than entering (*p* = 0.0171) and was unaffected by condition duration (*p* = 0.0647).

## Discussion

Here, we describe a simple technique that mitigates the nystagmus and powerful but temporary sensations of vertigo during entry into and exit from a high-field strength MRI scanner. Such side-effects of high-field strength MRI are more than a nuisance since they may cause patients who have recently undergone surgery to vomit and strain, which can have serious side effects (5). Using results from our model, we designed the experimental protocol to take advantage of innate central vestibular processing. Experimentally and mathematically, we found that increasing the duration of entry into and exit from a 7T MRI bore can blunt the nystagmus response, which had an associated reduction in the perceived intensity of vertigo. Furthermore, an entry duration of 2 min eliminated spinning vertigo symptoms in most participants, and an entry duration of 5 min eliminated all but the mildest vestibular symptoms. The model also supported that exit durations could be briefer than entry durations after (at least) 5 min inside the magnet because the post-peak was much smaller (Figure 1). Subjects also reported that the duration and intensity of symptoms were less on exiting the MRI bore, regardless of entry duration.

The blunted nystagmus response occurred upon entering the magnetic field because the brain adapted to a constant acceleration vestibular stimulus (29, 30). Furthermore, when in the supine position, linear acceleration from gravity helps to suppress the angular acceleration induced by MVS *via* the “rotational feedback” loop (22, 31). By better matching the duration of the participant's entry into the 7 Tesla magnetic field to the time constant of vestibular adaptation, we show that nystagmus and vertigo can be minimized on both entry and exit (Figures 3, 4). Previously, we had anecdotally noted a temporal correlation between the initial peak in nystagmus after entering the MRI and the duration of a participant's experience of vertigo (11). The findings here further support that the two occur at similar times as blunting this peak in nystagmus on entry and exit also mitigates vertigo (Figures 3B, 4A). It is unknown whether vertigo correlates with the peak or the slope of nystagmus SPV, but this could be further explored, as it has implications for magnetic fields above 7 Tesla, in which the nystagmus is expected to be much stronger (Figure 1C). Furthermore, entry into the MRI provides an easy way to manipulate changing acceleration stimulus, *i.e*., jerk. Altering the entry rate into a strong MRI can be systematically explored through MVS motion protocols to understand better how the vestibular system responds to different patterns of angular acceleration stimuli.

**Figure 5:**
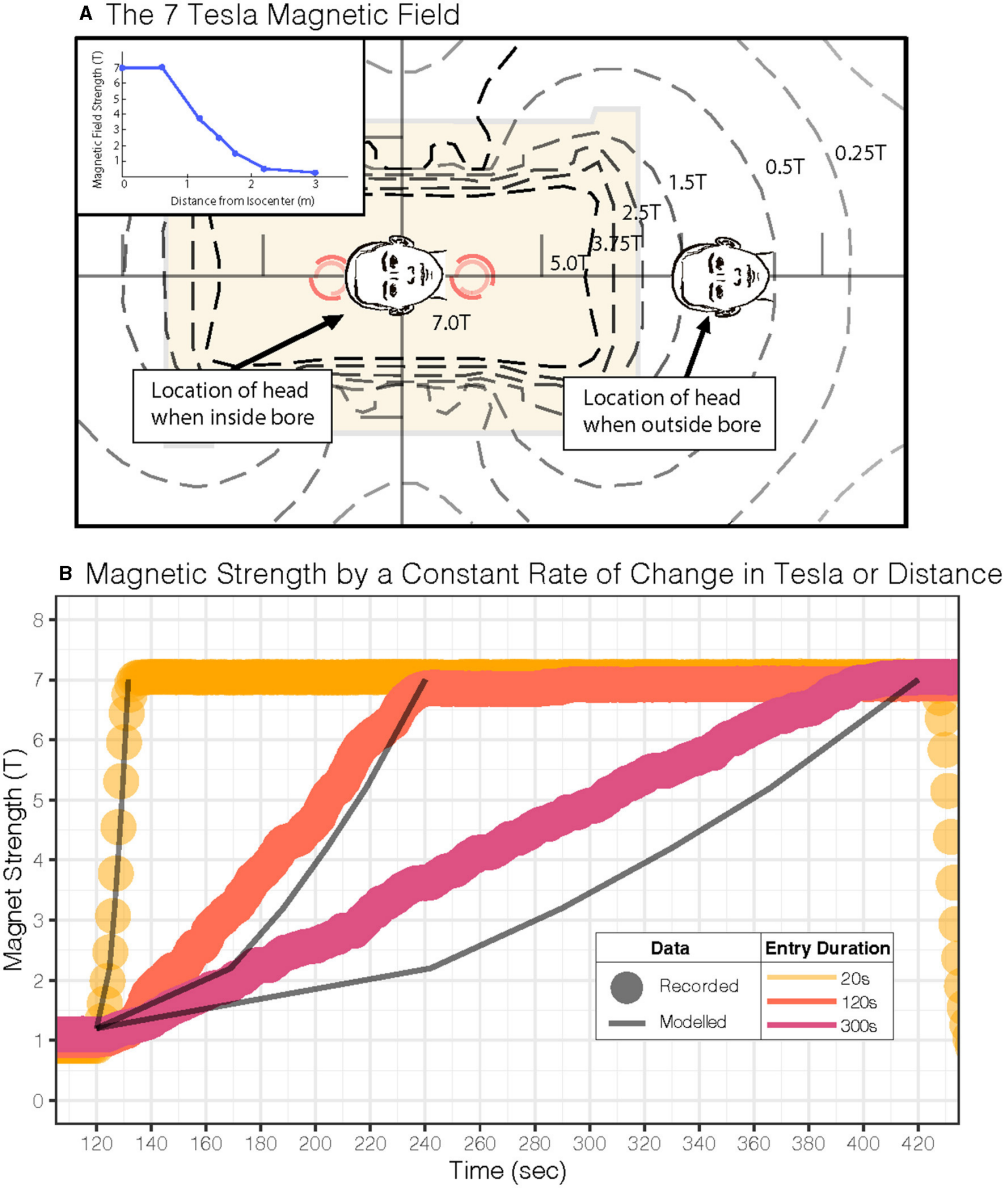
Magnetic field for our 7 Tesla MRI scanner and study parameters. **(A)** MRI magnetic field map for our scanner is shown. The inset shows the linear gradient of the head approaching the MRI bore isocenter. **(B)** Experimental magnetometer recordings during a constant Tesla/s profile (dots: •) and predictions (lines: —) for a constant table velocity entry into the MRI; each dot (•) represents one second of recorded experimental data, and the lines (—) represent predicted Tesla. Both approaches show a similar average rate of change in the magnetic field over time (see Table 1).

The results of this study provide a practical approach to minimizing vertigo in 7 T scanners and potentially harmful side effects (6). Our model and experimental design used a linearly increasing magnetic field strength, which, due to the non-linear fringe magnetic field of our MRI machine, necessitated a sigmoid velocity profile for the table entry into the MRI bore (Figure 5A). This protocol for table movement is currently not available on MRI scanner beds. While the transition through the magnetic field is steep during a short entry duration, this is not the case for longer-duration entries. Theoretically, we found that a constant table velocity at 0.05 T/s (duration: 120 s) and 0.02 T/s (duration: 300 s) shows a rate of change of magnetic field strength comparable to one that increases linearly with increasing magnetic field (Figure 5B; Table 1). Therefore, simply entering the MRI at a constant but lower rate may adequately suppress sensations of vertigo even at other (higher and lower) magnetic field strengths. Finally, the model supports the idea that the exit duration could be less than the entry duration since the peak amplitude of nystagmus after leaving the magnet is smaller and perceived vertigo less intense (Figures 1B, 3D, 4B).

Previous studies in MRI-induced dizziness also found that longer durations (~60 s) at lower entry velocities (0.05 m/s) into and out of the magnet can reduce sensations of vertigo (15). This was also observed in prior studies (32); however, at that time, the mechanism of MRI-induced dizziness was thought to be related to electromagnetic induction that, in theory, would also be reduced by slower entry (32). Since then, the most likely hypothesis that explains MRI-induced dizziness is a magnetohydrodynamic Lorentz force occurring in the inner ear (14). The current study supports the conclusion that lower entry rates can reduce vertigo symptoms but adds to our understanding of the mechanism. We show here that the threshold for vertigo perception and nystagmus profile depends on velocity into and out of the bore (rate of change in Tesla) in addition to the known effect of the absolute amplitude of the field (11, 33). Nevertheless, our studies reinforce that slower movement around the magnetic field reduces vertigo sensations albeit by an explanation relying on vestibular adaptation rather than electromagnetic induction.

Notably, the strategy presented here for mitigating vertigo does not eliminate the nystagmus or the brain's processing of the vestibular stimulus. Confounding effects of MVS during resting state functional MRI would still occur (34–37). Another way to mitigate both vertigo and nystagmus would be to pitch the head to the static position at which no nystagmus or vertigo arises (11). However, this “null” position varies among participants, and this solution is not possible with the restrictions of current radio-frequency signal receiver coils designed for head imaging. Furthermore, just keeping one's eyes fixed on a target while suppressing nystagmus SPV during MVS is inadequate to stop the underlying central vestibular activity within the MRI machine, which could still be a confound for functional MRI studies since the suppression of nystagmus is by fixation (38). Our study indicates that collecting functional MRI data in the first 5 min should be limited while vestibular adaptation is maximal—although future studies could focus on examining the loci of vestibular adaptation. The explanations for minimizing vertigo depend on the patient's head being within the isocenter of the magnetic field. In some clinical scans, patients are introduced into the scanner feet first, where the head may rest within a lower-strength magnetic field than the isocenter. Patients with their heads at this lower magnetic field strength may experience less vertigo (1). Future studies should further examine strategies to minimize the discomforting and potentially harmful side effects of MVS (2, 6, 7), especially as magnetic field strengths increase.

## Data availability statement

The datasets presented in this study can be found in online repositories. The names of the repository/repositories and accession number(s) can be found below: Figshare, https://doi.org/10.6084/m9.figshare.23632338.

## Ethics statement

The studies involving humans were approved by IRB-3, Johns Hopkins University School of Medicine. The studies were conducted in accordance with the local legislation and institutional requirements. The participants provided their written informed consent to participate in this study.

## Author contributions

JP: Conceptualization, Formal Analysis, Funding acquisition, Investigation, Methodology, Visualization, Writing – original draft, Writing – review & editing. AS: Formal Analysis, Methodology, Software, Validation, Writing – review & editing. DR: Formal Analysis, Investigation, Methodology, Software, Writing – review & editing. DZ: Conceptualization, Funding acquisition, Resources, Supervision, Validation, Writing – review & editing. JO-M: Formal Analysis, Methodology, Software, Validation, Writing—review & editing. BW: Conceptualization, Formal Analysis, Funding acquisition, Investigation, Methodology, Visualization, Writing—original draft, Writing—review & editing.
